# Beyond Uniform Impairment: Investigating Declarative Memory Profiles in Nonspecific Mild Intellectual Disability Using Latent Profile Analysis

**DOI:** 10.1111/jir.70039

**Published:** 2025-09-03

**Authors:** Urszula Sajewicz‐Radtke, Bartosz M. Radtke, Paweł Jurek, Michał Olech, Ariadna Łada‐Maśko

**Affiliations:** ^1^ Laboratory of Psychological and Educational Tests Gdańsk Poland; ^2^ Institute of Psychology University of Gdańsk Gdańsk Poland; ^3^ Department of Psychology Medical University of Gdańsk Gdańsk Poland

**Keywords:** cognitive profiles, declarative memory, high‐performing subgroup, intellectual disability, latent profile analysis, memory heterogeneity, TOMAL‐2

## Abstract

**Background:**

Significant memory impairments are consistently observed in individuals with intellectual disabilities (ID), but considerable variability exists. This study investigated the heterogeneity of declarative memory in children and adolescents with nonspecific mild intellectual disability (NSID) to identify distinct memory profiles and potential predictors of this disability.

**Methods:**

A latent profile analysis (LPA) was conducted on a large sample (*N* = 999, including 114 with NSID) using six supplementary memory indices from the Test of Memory and Learning—Second Edition (TOMAL‐2). A logistic regression analysis subsequently examined the predictive power of TOMAL‐2 indices for NSID diagnosis.

**Results:**

LPA revealed two distinct memory profiles: a ‘memory impaired group’ (24% of the total sample) with below‐average scores across all indices and a ‘high‐performers group’ (76%) with consistently above‐average scores. Individuals with NSID were significantly more likely to belong to the ‘memory impaired group’. Logistic regression analysis revealed that lower scores on the Attention/Concentration Index, Sequential Memory Index and Verbal Delayed Recall Index were the strongest predictors of NSID. However, notably, 25% of individuals with mild NSID were classified in the ‘high performers group’, exhibiting typical or above‐average memory scores across multiple indices.

**Conclusions:**

This study demonstrates significant heterogeneity in declarative memory among individuals with NSID, challenging the assumption of uniform impairment. The identified memory profiles and predictive indices offer valuable insights for more precise diagnostic assessment and the development of tailored interventions. Further research should investigate the factors contributing to this variability and explore the potential of these findings for improved support and educational strategies.

**Trial Registration:** NCT06215092

**Abbreviations**
ACI: Attention/Concentration Index
AIC: Akaike information criterion
ARI: Associative Recall Index
BIC: Bayesian Information Criterion
FRI: Free Recall Index
ID: intellectual disabilities
IQ: intelligence quotient
LI: Learning Index
LPA: latent profile analysis
MA: mental age
MALCP: minimum average latent class probabilities for most likely latent class membership
NSID: nonspecific mild intellectual disability
REPLIC: the best loglikelihood value has been replicated
SRI: Sequential Recall Index
TD: typically developing
TOMAL‐2: Test of Memory and Learning—Second Edition
VDRI: Verbal Delayed Recall Index

## 1 Background

Memory is a fundamental cognitive process that has been extensively studied in both healthy individuals and various clinical populations. Consistent findings indicate significant memory impairments among individuals with intellectual disabilities (ID) (van der Molen et al. 2009; Vicari and Carlesimo 2002; Vicari et al. 2016). However, the considerable variability in these deficits, both between and within individuals, raises questions about whether all memory components are equally affected or if specific aspects are disproportionately impacted. This heterogeneity suggests that atypical development in ID results in variable cognitive profiles, often described as ‘peaks and valleys’, reflecting both strengths and weaknesses (Edgin et al. 2010; Karmiloff‐Smith 2009; van der Molen et al. 2009; Sajewicz‐Radtke et al. 2022).

Explicit memory, which involves the conscious recall of past experiences, is typically assessed using recall and recognition tasks that require intentional retrieval of previously encoded information (Ober 2014; Kahana and Wagner 2024). This process relies on deliberate learning, strategic use of encoded information and effective retrieval strategies (Kump et al. 2015) and is influenced by an individual's general knowledge (Kump et al. 2015) as well as attentional resources (Naya 2022). The neurobiological underpinnings of these processes have been discussed elsewhere (Baddeley and Jarrold 2007; Roediger 2008; Sridhar et al. 2023). Vicari et al. (2016) emphasize that differential patterns of memory abilities exist across various etiological groups of individuals with ID. The focus of this research is on explicit memory, given its crucial role in learning and education for individuals with nonspecific ID (NSID), with the aim of characterizing explicit memory profiles to inform effective interventions.

The research explored indicates a complex interplay of various factors influencing memory performance in individuals with NSID. Notably, under favourable encoding and retrieval conditions, individuals with NSID can achieve memory performance comparable to that of typically developing (TD) peers, particularly when matched by mental age (MA) (Carlin et al. 2001; Dulaney and Ellis 1991). Optimal conditions for memory retention include the use of visual modalities during encoding, balanced presentation of old and new items in recognition tests, and the implementation of effective strategies supported by practice and prompting (Dulaney and Ellis 1991; Carlin et al. 2001; Lifshitz‐Vahav and Vakil 2014). Despite these conditions, free recall remains significantly impaired, particularly in auditory tasks, indicating a possible retrieval stage deficit that warrants further investigation (Dulaney and Ellis 1991). Furthermore, relationships between intelligence levels and memory performance show mixed results, with some studies finding correlations between IQ and memory aspects, whereas others do not consistently replicate these findings (Perrig and Perrig 1995; Lifshitz‐Vahav and Vakil 2014).

The encoding stage is critical in understanding memory performance, with research highlighting various factors affecting memory in individuals with NSID, including modality, strategy use, intentional versus incidental learning and practice effects (Lifshitz‐Vahav and Vakil 2014). Studies matching participants by MA have found that memory performance is preserved in visual tasks, whereas auditory tasks yielded better outcomes only when visual scaffolding was provided (Atwell et al. 2003). The effectiveness of strategy use varies between recognition and free recall tasks, with equal performances noted when using semantically related pictures (Dulaney and Ellis 1991) or precise procedural manipulations, such as fade‐in techniques (Carlin et al. 2001). Results concerning intentional and incidental learning are inconsistent; some studies report preserved memory under intentional conditions (Dulaney and Ellis 1991), whereas others highlight better performance in incidental conditions (Jones et al. 2002). Overall, practice and prompting have been shown to positively influence memory retention, reinforcing their importance in enhancing memory performance (Lifshitz‐Vahav and Vakil 2014). Additionally, in spatial memory tasks, individuals with NSID often underperform relative to MA‐matched peers, particularly in complex tasks requiring higher cognitive functions, emphasizing the need for tailored strategies in assessing memory capabilities in this population (Jones et al. 2002).

At the retrieval stage, recognition tests typically yield better outcomes compared to free recall tests, likely due to reduced demands on retrieval demands. Performance also varies between immediate and delayed recall conditions, suggesting potential weaknesses in long‐term memory consolidation (Gudjonsson and Henry 2003; Lifshitz‐Vahav and Vakil 2014). A distinctive observation in memory recall is the recency effect associated with short‐term memory, which is contrasted by a diminished or absent primacy effect, indicating relative weaknesses in long‐term memory compared to short‐term memory capabilities (Carlin et al. 2001; Carlesimo et al. 1997).

To our knowledge, this is the first study of this scope to comprehensively investigate a wide range of declarative memory using Latent Profile Analysis in children and adolescents from the general population and those with NSID. Previous research aimed at characterizing memory profiles in individuals with intellectual disabilities (ID) has primarily concentrated on working memory (Lifshitz et al. 2016; Roording‐Ragetlie et al. 2018; Schuchardt et al. 2010) and has often focused on specific conditions, such as Down syndrome (Baddeley and Jarrold 2007; Godfrey and Lee 2018; Næss et al. 2011), Williams syndrome (Martens et al. 2008; Mervis and John 2010; Serrano‐Juárez et al. 2023) or Fragile X syndrome (Schmitt et al. 2019). Additionally, existing research is often constrained by methodological limitations, including small clinical sample sizes, the inclusion of participants with varying degrees of disability and reliance on experimental methods for measuring memory (Vicari et al. 2016; Lifshitz‐Vahav and Vakil 2014).

The aim of the study is to investigate the characteristics of declarative memory in children and adolescents aged from 5 years to 19 years and 11 months and to examine whether individuals with NSID exhibit its specificity. This comprehensive exploration of explicit memory in individuals with NSID aims to contribute towards a better understanding of specific characteristics of their memory functioning. Furthermore, the research investigates whether the value of memory indicators can predict the presence of intellectual disabilities, thereby supporting the diagnostic process for assessing cognitive functioning in individuals with mild intellectual disability. Through these objectives, it may support the development of more effective interventions and educational strategies tailored to their specific needs. The use of psychometric methods to measure declarative memory (Test of Memory and Learning TOMAL‐2; Reynolds et al. 2024), along with current norms for the population, will allow for a better understanding of the differences and characteristics of memory profiles in individuals with NSID.

## 2 Methods

### 2.1 Participants and Procedure

This project was approved by the Ethics Board for Research Projects at the Medical University of Gdańsk, Poland (decision no. KB/749/2023‐2024).

#### 2.1.1 Nonclinical Sample

The sample consisted of 885 individuals (440 females and 503 males) aged 5–19 years and 11 months (*M* = 12.47; SD = 4.05). The study's participants, due to their diverse age range, were educated at a variety of institutions. Moreover, there was a broad spectrum in the study cohort that exhibited diversity in terms of their parents' educational levels. Data on the sample composition, categorized by the type of educational institution and encompassing the entire study cohort, is presented in Table 1.

**TABLE 1:** Demographic characteristics of the study sample: Overall and by normative sample versus intellectual disability groups.

Variable	Total	Normative sample	Intellectual disability
	*N* (%)	*N* (%)	*N* (%)
Gender			
Female	496 (50)	440 (50)	56 (49)
Male	503 (50)	445 (50)	58 (51)
Size of place of residence			
Less than 5000 residents	240 (24)	220 (25)	20 (18)
Between 5000 and 100 000 residents	297 (30)	255 (29)	42 (37)
More than 100 000 residents	462 (46)	410 (46)	52 (46)
Age group			
5:00–6:11	96 (10)	96 (11)	—
7:00–9:11	171 (17)	171 (19)	—
10:00–15:11	505 (51)	401 (45)	104 (91)
16:00–19:11	227 (23)	217 (25)	10 (9)
Mother's education			
Primary	39 (4)	20 (2)	19 (17)
Vocational	191 (19)	137 (15)	54 (47)
Secondary	241 (24)	208 (24)	33 (29)
Bachelor's/engineering	78 (8)	76 (9)	2 (2)
Master's degree	322 (32)	321 (36)	1 (1)
Postgraduate	39 (4)	39 (4)	—
Academic degree or Title	7 (1)	7 (1)	—
Missing data	82 (8)	77 (9)	5 (4)
Father's education			
Primary	47 (5)	24 (3)	23 (20)
Vocational	279 (28)	224 (25)	55 (48)
Secondary	240 (24)	213 (24)	27 (24)
Bachelor's/engineering	75 (8)	75 (8)	—
Master's degree	243 (24)	241 (27)	2 (2)
Postgraduate	14 (1)	14 (2)	—
Academic degree or title	7 (1)	7 (1)	—
Missing data	94 (9)	87 (10)	7 (6)
Educational institution			
Kindergarten	90 (9)	90 (10)	—
Primary school	577 (58)	510 (58)	67 (59)
Vocational school	67 (7)	67 (8)	—
Technical school	57 (6)	57 (6)	—
General secondary school (high school)	145 (15)	145 (16)	—
Special education school	50 (5)	3 (< 1)	47 (41)
Higher education institution	11 (1)	11 (1)	—
Missing data	2 (< 1)	2 (< 1)	—
Diagnosis (more than one possible)			
Developmental disorders	8 (1)	4 (< 1)	4 (4)
ADHD	31 (3)	30 (3)	1 (1)
ADD	1 (< 1)	1 (< 1)	—
Dyslexia	36 (4)	36 (4)	—
Dysgraphia	18 (2)	18 (2)	—
Dysorthography	34 (3)	34 (4)	—
Dyscalculia	18 (2)	18 (2)	—
Speech development disorders	5 (1)	—	5 (4)
Other	3 (< 1)	3 (< 1)	—

#### 2.1.2 Clinical Sample

The sample consisted of 114 individuals (56 females and 58 males) aged from 10 to 19 years and 11 months (*M* = 13.35; SD = 1.79) with a diagnosis of mild intellectual disability. All participants underwent a complete intellectual disability diagnostic process at state centres specializing in such assessments (see Appendix S1). They received an official document confirming the diagnosis and are enrolled in a special education programme, either in mainstream schools or in special schools. Only individuals without any additional diagnoses were included in the study. The research focused solely on individuals diagnosed with nonspecific mild intellectual disability.

#### 2.1.3 Procedure

All participants (clinical and nonclinical) underwent complete declarative memory assessment using the Polish version of the Test of Memory and Learning: Second Edition (TOMAL‐2; Reynolds et al. 2024) test by qualified diagnosticians from psychological and educational counselling centres in Poland in the years 2023–2024. Information about the research was sent to psychological and educational counselling centres all over Poland. Next, psychologists from the counselling centres that were interested in taking part in the research were trained in the research procedure. Subsequently, psychologists recruited children from schools and other educational institutions for the study. Parents were informed by the psychologist that the counselling centre takes part in the scientific research and then about the scope of the study and the data provided. After receiving the above information, the parents decided whether to consent to the child's participation in the study or not. Eventually, participants came from all over Poland, and the structure of the study participants corresponds to the structure of the country's population, taking into account regions and differentiation of the place of residence, as well as the parent's education level (see Table 1). The written consent to participate in the study was collected from all parents, whose children took part in the study. No sensitive personal data were gathered. No payment was provided for participation in the study.

### 2.2 Declaration on the Use of Artificial Intelligence Generated Content (AIGC)

We confirm that no Artificial Intelligence Generated Content (AIGC) tools, including ChatGPT or other large language models (LLMs), were used in the development of any portion of this manuscript. All content was created solely by the listed authors.

### 2.3 Measures

The TOMAL‐2 (Reynolds et al. 2024) arguably has the broadest range of memory tasks available in a standardized memory battery. The core battery of the TOMAL‐2 consists of eight subtests, divided into four verbal and four nonverbal tasks. These subtests are used to calculate the Verbal Memory, Nonverbal Memory and Composite Memory indexes (Appendix S2). The core battery provides a comprehensive evaluation of memory functions, including free and associative recall, meaningful and abstract memory, sequential recall and the learning process. This set of eight subtests addresses most questions related to memory assessment. In addition to the core battery, the TOMAL‐2 includes four verbal and two nonverbal supplementary subtests (Appendix S2), designed to offer a more detailed analysis of memory. These supplementary subtests are particularly useful for neuropsychologists and researchers. By incorporating supplementary subtests, additional memory indexes can be derived, such as the Verbal Delayed Recall Index (VDRI), Attention/Concentration Index (ACI), Sequential Recall Index (SRI), Free Recall Index (FRI), Associative Recall Index (ARI) and Learning Index (LI; Appendix S2). A Verbal Delayed Recall Index (VDRI) can also be calculated by administering a delayed recall procedure for two verbal subtests from the core battery. All TOMAL‐2 index scores are standardized (*M* = 100, SD = 15), with possible values typically ranging from 40 to 160. The scoring metric is analogous to that used for IQ scores, with higher values indicating better memory performance.

### 2.4 Statistical Analysis

To address the research questions, two complementary analytical approaches were employed: Latent Profile Analysis (LPA; Collins and Lanza 2010; Muthén 2002) and logistic regression analysis (Hilbe 2009). An LPA was conducted to explore memory profiles among children and adolescents aged 5–19 (RQ1) and to investigate the specific memory functioning of individuals with mild intellectual disability (ID) (RQ2). The analysis was based on a sample of *N* = 999, which included 114 individuals diagnosed with mild ID. LPA was performed using six supplementary indices derived from the TOMAL‐2 test: Verbal Delayed Recall Index (VDRI), Attention/Concentration Index (ACI), Sequential Recall Index (SRI), Free Recall Index (FRI), Associative Recall Index (ARI) and Learning Index (LI). The LPA model was estimated using flexible assumptions, allowing variances and covariance matrices to differ across profiles. To ensure the algorithm reached the global and local minimum of the maximum likelihood estimator, the model fitting procedure employed 1000 random starting values.

Models were tested sequentially, ranging from two to six latent profiles. Model selection was guided by the following criteria (see Weller et al. 2020): the Bayesian Information Criterion (BIC), where smaller values indicate a better fit to the data; profile entropy, with higher values (range: 0–1) indicating better separation between profiles; the minimum average latent class probabilities for the most likely latent class membership (range: 0–1), reflecting the quality of profile assignment; and the stability of the Akaike Information Criterion (AIC) across models fitted with random starting values. Finally, models with profiles representing less than 5% of the sample were rejected.

The optimal model was chosen based on these criteria and further evaluated using a chi‐square test to examine whether individuals with mild ID were significantly more likely to belong to specific latent profiles compared to their peers without ID. This approach allowed for the identification of meaningful groupings in memory functioning, examined their association with intellectual disability diagnosis, and provided a statistical analysis of the relationship between ID diagnosis and profile membership, addressing RQ2.

To assess whether memory indices predict the likelihood of an ID diagnosis (RQ3), a backward logistic regression analysis was conducted. For this analysis, the 114 participants with mild ID were matched with an equal‐sized comparison group from the general population, ensuring equivalence in age (in years), gender and place of residence. The outcome variable was the presence of an ID diagnosis, whereas all nine TOMAL‐2 indices (general, nonverbal, verbal and six supplementary indices) served as predictors. Logistic regression was selected for its ability to model binary outcomes probabilistically based on continuous predictors, providing a direct answer to RQ3.

Additionally, independent samples *t* tests with Cohen's *d* effect size calculations were performed to compare TOMAL‐2 indices between the two groups. These analyses provided insights into the magnitude of group differences, supplementing hypothesis testing with measures of practical significance to enhance the interpretability of the results.

The calculations were performed using Mplus 8.11 (Muthen and Muthen 2017) and the R environment (Team 2024).

## 3 Results

### 3.1 Latent Profile Analysis (RQ1 and RQ2)

**FIGURE 1:**
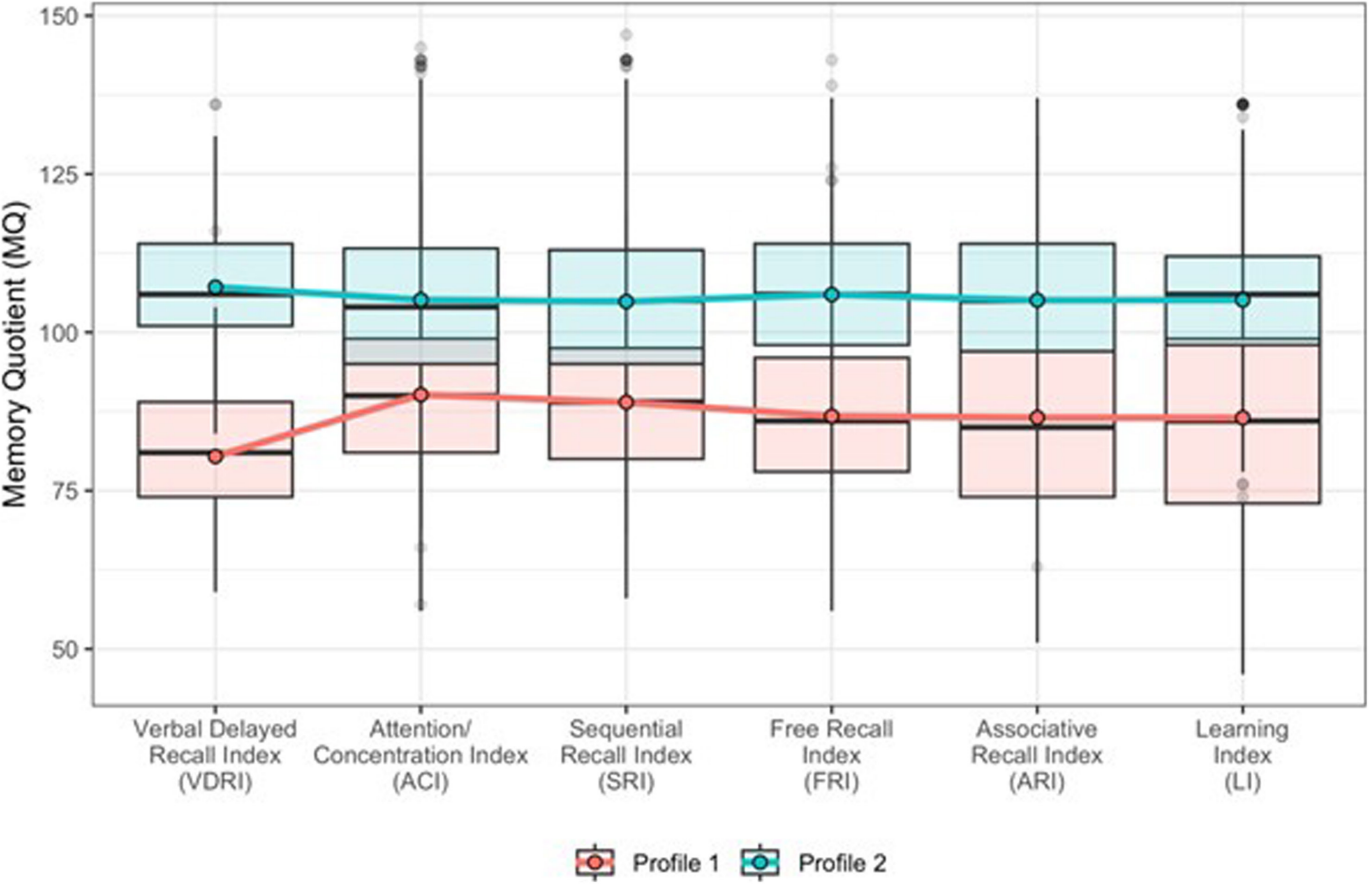
Distributions of standardized scores from the TOMAL‐2 test across identified latent profiles.

As shown in Table 2, the Latent Profile Analysis (LPA) identified an optimal model with two latent profiles based on the six supplementary indices from the TOMAL‐2 test (standardized scores). Model fit indices indicated that the two‐profile solution provided the best fit, supported by the lowest Bayesian Information Criterion (BIC = 44 685) and high profile's entropy (0.74), which ensured adequate classification accuracy. Profile 1 (Memory Impaired Group) accounted for 24% of the sample and exhibited below‐average scores across all indices, particularly on the VDRI. In contrast, Profile 2 (High Performers Group) comprised 76% of the sample and displayed consistently above‐average scores on all indices. Figure 1 presents the distributions of standardized scores from the TOMAL‐2 test (six supplementary indices) within the identified latent profiles.

**TABLE 2:** Model fit indices and characteristics of latent profiles identified based on standardized scores from the TOMAL‐2 test.

Model	AIC	BIC	Final class proportions for the latent classes based on their most likely latent class membership						Entropy	MALCP	REPLIC
			p1	p2	p3	p4	p5	p6			
2 profiles	44 415	44 685	0.24	0.76					0.74	0.90	Yes
3 profiles	44 289	44 696	0.02	0.21	0.77				0.85	0.90	No
4 profiles	44 207	44 751	0.25	0.57	0.02	0.16			0.77	0.84	No
5 profiles	model not converge		0.24	0.02	0.44	0.28	0.02		0.76	0.81	No
6 profiles			0.03	0.15	0.01	0.03	0.73	0.05	0.91	0.85	No

Abbreviations: MALCP, minimum average latent class probabilities for most likely latent class membership; REPLIC, the best loglikelihood value has been replicated.

### 3.2 Association With Intellectual Disability

Chi‐square analysis revealed a significant association between intellectual disability diagnosis and profile membership, $\chi^{2}$ (1, *N* = 999) = 189.55, *p* < 0.001. Individuals with mild intellectual disability were more frequently represented in Profile 1 (memory impaired group), with 75% (*n* = 86) of participants diagnosed with mild ID in this profile, compared to only 25% (*n* = 28) in Profile 2 (high performers group). These findings indicate that individuals with mild ID predominantly exhibit deficits across all memory dimensions, particularly on the VDRI. In contrast, the normative sample showed an opposite pattern: 17% (*n* = 149) of this group were assigned to Profile 1, whereas 83% (*n* = 736) were classified in Profile 2, characterized by balanced performance across all six TOMAL‐2 indices. However, the remaining 25% (*n* = 28) of individuals with mild ID were assigned to the high performers group. Although limited by the small sample size and lack of subgroup‐level demographic information, this analysis revealed that these individuals displayed average to above‐average scores across all indices. The strongest relative performance was observed on the Learning Index and Associative Recall Index, indicating relatively preserved capacity for acquiring and integrating new information, as well as for forming associations. This subgroup challenges the assumption of uniformly low memory performance in individuals with mild ID and highlights the importance of individualized assessment.

### 3.3 Logistic Regression Analysis (RQ3)

Logistic regression analysis was conducted to evaluate the predictive power of the TOMAL‐2 indices in identifying intellectual disability. The nine indices (three general and six supplementary) were included as predictors, with the presence of a mild ID diagnosis serving as the outcome variable. A backward selection procedure was applied to refine the model. Table 3 presents the results for both the baseline model (including all predictors) and the final model (resulting from the backward selection). Both logistic regression models were statistically significant and accounted for 76% and 75% of the variance in the ID diagnosis, respectively (as indicated by Nagelkerke *R* ^2^ in Table 3). The final model achieved a classification accuracy of 90%. These results indicate that lower scores on the ACI, SMI and DVRI indices were the strongest predictors of an intellectual disability diagnosis.

**TABLE 3:** Predictive performance of baseline and final logistic regression models for mild intellectual disability diagnosis.

Predictor	Baseline model (all predictors)			Final model (resulting from backward selection)		
	*B*	SE	*p* value	*B*	SE	*p* value
Intercept	21.41	7.75	< 0.01	21.81	2.81	< 0.01
General Memory Index	−0.19	0.63	0.77	—	—	—
Verbal Memory Index	0.15	0.37	0.69	—	—	—
Nonverbal Memory Index	0.06	0.35	0.87	—	—	—
Verbal Delayed Recall Index	−0.03	0.02	0.23	—	—	—
Attention/Concentration Index	−0.16	0.06	< 0.01	−0.10	0.03	< 0.01
Sequential Recall Index	0.07	0.06	0.26	—	—	—
Free Recall Index	−0.04	0.07	0.58	−0.07	0.02	< 0.01
Associative Recall Index	−0.07	0.04	< 0.05	−0.07	0.02	< 0.01
Learning Index	−0.01	0.05	0.80	—	—	—
Model fit and performance metrics						
Nagelkerke *R* ^2^	0.76			0.75		
Accuracy	0.91			0.90		
Precision	0.90			0.89		
Recall	0.93			0.92		
Specificity	0.89			0.89		

### 3.4 Group Comparisons

Independent sample *t* tests (see Table 4 for details) revealed significant differences across all nine TOMAL‐2 indices between the mild ID group and the matched comparison group (*t* values ranged from −10.97 to −16.38, all *p* < 0.01). Effect sizes (Cohen's *d*) ranged from 1.91 (Learning Index) to 2.53 (General Memory Index), indicating large differences in memory performance. In addition to means and standard deviations, Table 4 also reports minimum and maximum values, as well as the 10th and 90th percentiles (Q10 and Q90), for each index and group. These descriptive statistics illustrate the broader distribution of scores and show that individuals with mild ID consistently scored lower across all indices, with their Q90 values generally not substantially exceeding the comparison group's Q10. This pattern highlights the substantial and consistent performance gap between groups and provides insight into the extent to which ceiling or floor effects may have occurred.

**TABLE 4:** Group comparisons of TOMAL‐2 indices: Mild ID group versus matched comparison group.

Variable	Mild ID group (*n* = 114)			Comparison group (*n* = 114)			*t*	*p*	*d*
	*M* ± SD	Min–Max	Q10–Q90	*M* ± SD	Min–Max	Q10–Q90			
General Memory Index	79.50 ± 10.44	58–105	65–93	105.99 ± 13.75	61–140	90–121	−16.38	< 0.01	2.53
Verbal Memory Index	82.13 ± 12.25	51–109	66–98	105.68 ± 13.75	63–138	88–121	−13.65	< 0.01	2.28
Nonverbal Memory Index	81.09 ± 10.07	59–105	68–95	104.98 ± 13.16	64–134	90–120	−15.39	< 0.01	2.13
Verbal Delayed Recall Index	86.14 ± 14.03	59–116	70–103	105.56 ± 12.67	59–131	94–120	−10.97	< 0.01	2.24
Attention/Concentration Index	83.99 ± 9.77	56–113	73–95	105.85 ± 13.65	69–143	91–125	−13.90	< 0.01	2.25
Sequential Recall Index	82.73 ± 10.23	58–122	71–95	105.11 ± 14.23	65–143	88–122	−13.64	< 0.01	2.22
Free Recall Index	81.51 ± 10.46	58–106	68–94	105.15 ± 12.49	64–131	90–118	−15.49	< 0.01	2.35
Associative Recall Index	82.50 ± 11.89	51–105	68–97	105.73 ± 12.48	68–131	91–120	−14.38	< 0.01	2.29
Learning Index	80.46 ± 14.21	51–111	61–98	104.60 ± 14.07	61–136	86–121	−12.89	< 0.01	1.91

*Note:* All indices are standardized scores from the TOMAL‐2 (*M* = 100, SD = 15), with higher values indicating better memory performance.

Abbreviations: *d*, Cohen's *d* effect size; *M* ± SD, mean and standard deviation; Min–Max, observed minimum and maximum values; *p*, significance level; Q10–Q90, 10th and 90th percentiles, representing the central range of scores; *t*, *t* value from independent samples *t* test.

## 4 Discussion

This study reveals significant heterogeneity in declarative memory performance among individuals with NSID, a finding that challenges the previously held assumption of uniform memory deficits within this population. The identification of two distinct latent profiles—a ‘memory impaired group’ and a ‘high‐performers group’—underscores the complexity of cognitive functioning in NSID. The substantial proportion (75%) of the NSID group falling within the ‘memory impaired group’ confirms memory impairment as a prominent feature of NSID, consistent with previous research (van der Molen et al. 2009; Vicari et al. 2016). However, the presence of a substantial ‘high‐performers group’ (25%) highlights the significant variability in memory abilities and challenges the notion of a homogenous deficit. This subgroup of individuals with mild ID demonstrated relatively strong performance across all six supplementary TOMAL‐2 indices. In particular, the Learning Index and Associative Recall Index were areas where this group tended to perform especially well, suggesting relative strengths in the ability to form and retain new associations. Although detailed demographic or clinical information for this subgroup was not available, their presence underscores the need for a more differentiated approach in both diagnostic evaluation and intervention planning for individuals with ID.

The logistic regression analysis successfully identified specific TOMAL‐2 indices (ACI, SMI and DVRI) as robust predictors of NSID. These findings hold significant implications for clinical assessment, suggesting these indices could improve diagnostic accuracy. However, the model's explained variance (75%) indicates that other factors, currently unidentified, contribute substantially to the development of NSID. Future research should investigate these additional factors, considering potential influences such as aetiology, comorbidities, environmental factors and the interaction between genetic and environmental influences.

The inconsistent findings in the literature regarding correlations between IQ and memory performance (Lifshitz‐Vahav and Vakil 2014; Perrig and Perrig 1995) are further contextualized by this study's demonstration of heterogeneous memory profiles within the NSID population. Our findings suggest that relying solely on IQ as a predictor of memory ability is insufficient, underscoring the value of a more nuanced, profile‐based approach to assessment. This profile‐based approach recognizes the unique cognitive strengths and weaknesses of each individual, potentially leading to more effective and tailored interventions.

The observed retrieval‐stage deficits (evidenced by lower VDRI scores and a recency effect without primacy) align with previous research suggesting weaknesses in long‐term memory consolidation among individuals with ID (Gudjonsson and Henry 2003; Lifshitz‐Vahav and Vakil 2014). The influence of factors such as encoding strategies and modality on memory performance further underscores the need for interventions that incorporate these factors.

Several limitations of this study warrant consideration when interpreting the results. First, the age range of the clinical sample (10–19 years and 11 months) may limit the generalizability of findings, as significant developmental changes in memory occur during this period. Restricting the sample to this age range potentially overlooks developmental influences on memory in individuals with NSID. Future research should incorporate a broader age range and, crucially, adopt a longitudinal design to track memory development over time and understand the dynamic evolution of memory profiles across the lifespan. Second, the study's sample primarily comprises individuals from Poland, a country with relatively high cultural and ethnic homogeneity, which limits the generalizability of findings to diverse cultural contexts. Replicating this research in other cultural settings with varied populations is crucial to assess the universality of the observed memory profiles. Third, the focus on individuals with only mild intellectual disability limits the scope of this research and prevents generalization to those with moderate or severe impairments. Future studies should expand to encompass individuals across the full spectrum of intellectual disability severity, as this may reveal distinct memory profiles and potentially highlight unique cognitive strengths and weaknesses not observed in the current study. In summary, although this study provides valuable insights, future research incorporating broader age ranges, longitudinal designs, diverse cultural populations and a wider range of intellectual disability severities is essential to enhance the generalizability of findings and comprehensively understand declarative memory in individuals with NSID.

## 5 Conclusions

This study demonstrates significant heterogeneity in declarative memory among individuals with nonspecific mild intellectual disability (NSID), challenging the assumption of uniform impairment. Two distinct memory profiles were identified: a ‘memory impaired group’ exhibiting widespread deficits and a ‘high‐performers group’ showing overall strong performance. Individuals with NSID were significantly overrepresented in the ‘memory impaired group’, highlighting memory impairment as a characteristic of NSID. However, a substantial proportion of individuals with NSID demonstrated typical or superior memory functioning. Specific memory indices (ACI, SMI and DVRI) proved strong predictors of NSID diagnosis, suggesting their potential utility in clinical assessment. Retrieval‐stage deficits, indicated by better recognition than recall performance and a recency effect without primacy, were also evident. Future research should utilize larger, more diverse samples and incorporate multiple assessment tools to enhance generalizability and deepen understanding of memory profiles in NSID. Further investigation into the factors underlying the observed heterogeneity is crucial to inform more effective interventions and support for individuals with NSID.

## Ethics Statement

This study was performed in line with the principles of the Declaration of Helsinki. Approval was granted by the Ethics Board for Research Projects at the Medical University of Gdańsk, Poland (decision no. KB/749/2023‐2024). The protocol of this study has been registered at https://clinicaltrials.gov/, registration number: NCT06215092.

## Conflicts of Interest

The authors declare no conflicts of interest.

## Supporting information


**Appendix S1:** Description of the procedure for assessing intellectual disability in Poland.

**Appendix S2:** Test of Memory and Learning – Second Edition (TOMAL‐2).

## Data Availability

The datasets used and analysed during the current study are available from the corresponding author on reasonable request.
