# Supplementary material for: Beyond Uniform Impairment: Investigating Declarative Memory Profiles in Nonspecific Mild Intellectual Disability Using Latent Profile Analysis
Source: J Intellect Disabil Res. 2025 Sep 3;69(12):1425–34. doi: 10.1111/jir.70039 (PMC12580476; doi:10.1111/jir.70039)
Supplement: Supplementary file 1 — Appendix S1: Description of the procedure for assessing intellectual disability in Poland. [file JIR-69-1425-s002.docx]

**Appendix 1**

**Description of the procedure for assessing intellectual disability in Poland**

The diagnosis of intellectual disability in Poland is a structured and multi-stage process that requires the involvement of various professionals, educators, and the child’s parents or guardians. The goal is to comprehensively assess the child's cognitive abilities, adaptive behaviors, and overall functioning to ensure appropriate support and intervention.

The process typically begins when difficulties are first noticed by parents, teachers, or pediatricians. Parents or guardians may observe that their child struggles with learning, problem-solving, or daily activities, while teachers in preschools or schools may identify developmental delays or difficulties in acquiring new skills. Pediatricians may also raise concerns about a child's development during routine check-ups. Once such concerns arise, the child is referred to a specialized diagnostic center where a comprehensive assessment takes place.

A team of at least three specialists is involved in the evaluation process, including a psychologist, an educational specialist, and a physician. Depending on the child’s needs, additional professionals such as speech therapists or physiotherapists may also participate. The first step in the diagnostic procedure is an IQ assessment conducted by a qualified psychologist with extensive training in psychometric testing. In Poland, only a psychologist who has completed a five-year master's degree and additional training in the application of intelligence assessment methods for special groups is authorized to conduct such evaluations. The most used test in Poland for assessing intellectual disability is the Stanford-Binet Intelligence Scales, Fifth Edition (Roid et al., 2017).

The next stage involves an evaluation of adaptive behaviors by an educational specialist, who interviews parents or guardians and reviews documentation to determine the child's ability to function in everyday life. The educational specialist responsible for assessing adaptive behaviors must also have completed a five-year master’s degree and possess a specialization in working with children with special educational needs or with intellectual disabilities.

A crucial part of the diagnosis is the functional assessment, which consists of several components. Observations of the child’s behavior, peer interactions, and preparedness for activities are documented. Parents or guardians provide essential information about the child's health, social development, and potential challenges. Educators and other professionals working with the child contribute insights into their difficulties, past interventions, and any additional support received. School and medical records are also reviewed to build a complete profile.

The diagnostic team also typically includes a medical specialist in psychiatry or neurology, who evaluates any potential medical conditions that might influence the child’s cognitive and adaptive functioning.

Finally, all gathered information is presented to a multidisciplinary evaluation committee, which includes the diagnosing specialists, parents, and a medical expert. Based on the collected data, the committee determines whether the child has an intellectual disability and assesses its severity. Below, we present a structured overview of this process.

**Flowchart: Diagnosis of Mild Intellectual Disability in Poland:**

1. **Initial Identification of Difficulties:**

- Parents or guardians recognize developmental or learning difficulties;
- Teachers in preschool or school observe potential intellectual or adaptive issues;
- Pediatricians identify possible developmental concerns during medical check-ups.

1. **Referral to Specialized Diagnostic Centers:**

- The child is directed to a center specializing in psychometric and functional assessments.
- The diagnostic process begins with a team of at least three specialists:
  - Psychologist;
  - Educational specialist;
  - Additional specialists (e.g., speech therapist, physiotherapist, physician) if required.

1. **Diagnostic Process:**

- **Step 1: IQ Assessment**
  - Conducted by a qualified psychologist with a master's degree (5 years) and additional certification in special group IQ testing;
  - Standardized intelligence tests are administered to evaluate cognitive functioning.
- **Step 2: Assessment of Adaptive Behaviors**
  - Conducted by a qualified educational specialist with a master's degree (5 years) and additional certification in working with children with special educational needs or with intellectual disabilities;
  - Evaluation based on interviews with caregivers, documentation review, and assessment of adaptive skills.
- **Step 3: Functional Diagnosis**
  - **Child Observation:**
    - Behavioral analysis;
    - Peer and adult interactions;
    - Preparedness for activities and cognitive skills;
    - Personal and emotional challenges.
  - **Parental/Caregiver Interview:**
    - Child’s health condition, behavioral and educational concerns;
    - Risk of social maladjustment;
    - Family, social, and economic status.
  - **Input from Other Specialists:**
    - Therapy directions and intervention history;
    - Types of existing disorders and previous support received.
  - **Review of Educational and Medical Documentation:**
    - Reports from psychological-pedagogical counseling centers;
    - Specialist assessments from hospitals, clinics;
    - School records, evaluation sheets.

1. **Final Decision by the Evaluation Committee:**

- A meeting involving:
  - Diagnostic specialists;
  - Specialist physician.
- A collective decision is made regarding the intellectual disability diagnosis and its severity level based on all gathered data.

ROID, G.H.; SAJEWICZ-RADTKE, U.; RADRTKE, B.M.; LIPOWSKA, M. 2017. *Skala Inteligencji Stanford-Binet, Edycja Piąta [Stanford-Binet Intelligence Scales, Fifth Edition]*, Gdańsk, Pracownia Testów Psychologicznych i Pedagogicznych [Laboratory of Psychological and Pedagogical Tests].

ROZPORZĄDZENIE MINISTRA EDUKACJI NARODOWEJ [REGULATION OF THE MINISTER OF NATIONAL EDUCATION]. 2017. *Rozporządzenie Ministra Edukacji Narodowej z dnia 7 września 2017 r. w sprawie orzeczeń i opinii wydawanych przez zespoły orzekające działające w publicznych poradniach psychologiczno-pedagogicznych
– z późniejszymi zmianami [Regulation of the Minister of National Education of 7 September 2017 on rulings and opinions issued by adjudicating teams operating in public psychological and educational counseling centers -* *with subsequent amendments ]***,** Dz.U. 2023 poz. 2061.
