# Supplementary material for: Beyond Uniform Impairment: Investigating Declarative Memory Profiles in Nonspecific Mild Intellectual Disability Using Latent Profile Analysis
Source: J Intellect Disabil Res. 2025 Sep 3;69(12):1425–34. doi: 10.1111/jir.70039 (PMC12580476; doi:10.1111/jir.70039)
Supplement: Supplementary file 2 — Appendix S2: Test of Memory and Learning – Second Edition (TOMAL‐2). [file JIR-69-1425-s001.docx]

**APPENDIX 2**

**Test of Memory and Learning – Second Edition (TOMAL-2)**

The core battery of the TOMAL-2 consists of eight subtests divided into four verbal and four nonverbal tasks. These subtests were used to calculate Verbal Memory, Nonverbal Memory, and Composite Memory indices (Table 1). The core battery provides a comprehensive evaluation of memory functions including free and associative recall, meaningful and abstract memory, sequential recall, and learning. This set of eight subtests addresses most questions related to memory assessment. In addition to the core battery, TOMAL-2 includes four verbal and two nonverbal supplementary subtests (Table 1) designed to offer a more detailed analysis of memory. These supplementary subtests are particularly useful to neuropsychologists and researchers. By incorporating supplementary subtests, additional memory indices such as Attention/Concentration, Sequential Recall, Free Recall, Associative Recall, and Learning can be derived (Table 2). A verbal delayed-recall index can also be calculated by administering a delayed-recall procedure for two verbal subtests from the core battery.

**Table 1.** Core and Supplementary Subtests and Indexes Available for the TOMAL-2

| **TOMAL-2** | **Core Subtests** | *M* | *SD* |
| --- | --- | --- | --- |
| *Verbal* | Memory for Stories | 10 | 3 |
|  | Word Selective Reminding | 10 | 3 |
|  | Object Recall | 10 | 3 |
|  | Paired Recall | 10 | 3 |
| *Nonverbal* | Facial Memory | 10 | 3 |
|  | Abstract Visual Memory | 10 | 3 |
|  | Visual Sequential Memory | 10 | 3 |
|  | Memory for Location | 10 | 3 |
| **Supplementary Subtests** | | | |
| *Verbal* | Digits Forward | 10 | 3 |
|  | Letters Forward | 10 | 3 |
|  | Digits Backward | 10 | 3 |
|  | Letters Backward | 10 | 3 |
| *Nonverbal* | Visual Selective Reminding | 10 | 3 |
|  | Manual Imitation | 10 | 3 |
| **Summary Scores** | | | |
| *Core Indexes* | Verbal Memory Index | 100 | 15 |
|  | Nonverbal Memory Index | 100 | 15 |
|  | Composite Memory Index | 100 | 15 |
| *Supplementary Indexes* | Sequential Recall Index | 100 | 15 |
|  | Free Recall Index | 100 | 15 |
|  | Associative Recall Index | 100 | 15 |
|  | Learning Index | 100 | 15 |
|  | Attention/Concentration Index | 100 | 15 |
|  | Verbal Delayed Recall Index | 100 | 15 |

**Table 2.** TOMAL-2 Indexes and Their Subtest Composition

| **INDEX** | **SUBTEST** |
| --- | --- |
| **Core Composite Indexes** | |
| *Verbal Memory* | Memory for Stories |
|  | Word Selective Reminding |
|  | Object Recall |
|  | Paired Recall |
| *Nonverbal Memory* | Facial Memory |
|  | Abstract Visual Memory |
|  | Visual Sequential Memory |
|  | Memory for Location |
| **Supplementary Composite Indexes** | |
| Verbal Delayed Recall | Memory for Stories Delayed |
|  | Word Selective Reminding Delayed |
| Attention/Concentration | Digits Forward |
|  | Letters Forward |
|  | Manual Imitation |
|  | Digits Backward |
|  | Letters Backward |
| Sequential Recall | Visual Sequential Memory |
|  | Digits Forward |
|  | Letters Forward |
|  | Manual Imitation |
| Free Recall | Facial Memory |
|  | Abstract Visual Memory |
|  | Memory for Location |
| Associative Recall | Memory for Stories |
|  | Paired Recall |
| Learning | Word Selective Reminding |
|  | Object Recall |
|  | Paired Recall |
|  | Visual Selective Reminding |
